# Bringing enzymes to the proximity party

**DOI:** 10.1039/d3cb00084b

**Published:** 2023-09-29

**Authors:** Gabrielle S. Tender, Carolyn R. Bertozzi

**Affiliations:** a Stanford University, Department of Chemistry and Sarafan ChEM-H Stanford CA 94305 USA; b Howard Hughes Medical Institute Stanford CA 94305 USA bertozzi@stanford.edu

## Abstract

Enzymes are used to treat a wide variety of human diseases, including lysosomal storage disorders, clotting disorders, and cancers. While enzyme therapeutics catalyze highly specific reactions, they often suffer from a lack of cellular or tissue selectivity. Targeting an enzyme to specific disease-driving cells and tissues can mitigate off-target toxicities and provide novel therapeutic avenues to treat otherwise intractable diseases. Targeted enzymes have been used to treat cancer, in which the enzyme is either carefully selected or engineered to reduce on-target off-tumor toxicity, or to treat lysosomal storage disorders in cell types that are not addressed by standard enzyme replacement therapies. In this review, we discuss the different targeted enzyme modalities and comment on the future of these approaches.

## Abstract 2

Targeted enzymes can drive cell type-selective reactions that are not yet possible with other therapeutic approaches.

## Graphical abstract

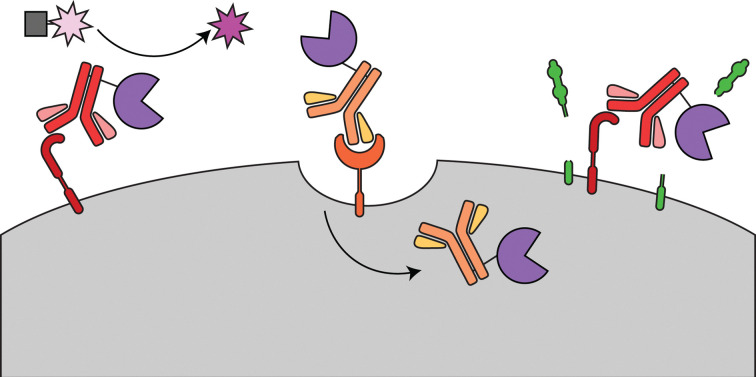

## 1. Introduction

Bifunctional therapeutics, including bispecific antibodies, molecular glues, and proteolysis targeting chimeras (PROTACs), are revolutionizing the therapeutic space. These modalities work by bringing two biomolecules or cells together, thereby driving novel interactions and reactions, such as directing immune cells to specific target cells or targeted protein degradation. Molecular glues and PROTACs promise to expand the druggable proteome by promoting degradation of proteins of interest whose disease-driving functions cannot be inhibited by small molecules or blocked by antibodies. These bifunctional molecules direct a ubiquitin ligase to a protein of interest, which induces its ubiquitination and initiates a cascade that leads to its eventual degradation by the proteasome.^1^ Recently this approach has expanded beyond ubiquitination to include other proximity-induced enzymatic reactions, including protein de-ubiquitination for stabilization,^2^ phosphorylation,^3^ and dephosphorylation.^4^

Current bifunctional therapeutics are fundamentally limited to the chemistries that our enzymes or cells can inherently perform in the same subcellular location as the enzymatic target. This reaction space can be expanded both by targeting human enzymes to non-endogenous locations or by directing non-human enzymes to disease-driving cells or macromolecules. These targeted enzymes offer a unique approach to further expand the druggable space beyond what is possible with other bifunctional therapeutics. In some recent examples, enzymes have been engineered and targeted to modify cell surfaces of only disease-driving cells.^5,6^ This approach applies the lessons of proximity-induced reactions from other bifunctional therapeutics: the enzymes have low activity while circulating the body, and the high local molarity enabled by the targeting moiety drives the reaction on only target cells.

**Fig. 1:**
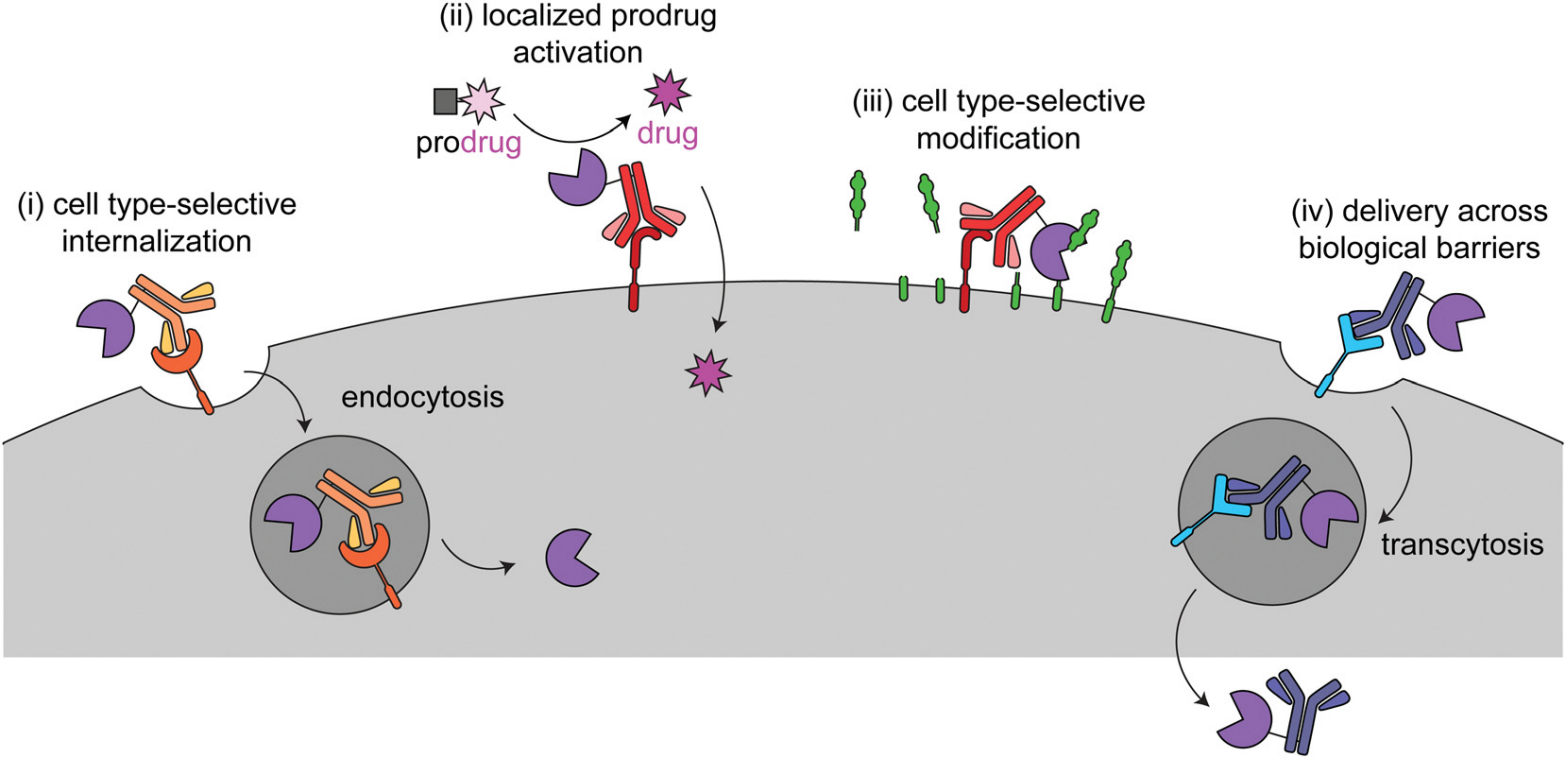
Overview of the four different targeted enzyme therapeutic approaches. In (i), the enzyme is targeted to a cell type-selective, internalizing receptor for intracellular delivery. This method is employed for cytotoxic enzymes in cancer (immunotoxins) and cell type-specific enzyme replacement therapy. In (ii) and (iii), the enzyme is targeted to a cell type-selective, non-internalizing receptor for prodrug activation or to catalyze modifications at the cell surface. (ii) has been used for antibody-dependent prodrug activation for cancer treatment. (iii) has been used to remove tumor-progressive sialic acids and mucins and for cleavage of inorganic pyrophosphate in Pompe disease. In (iv) the enzyme is targeted to a receptor for transcytosis across a biological barrier. This has been used to deliver therapeutic enzymes to the central nervous system.

This review focuses on current targeted enzyme strategies, including enzyme replacement therapies (ERT) and those that rely on proximity-induced reactions. There are four general approaches for current targeted enzyme therapies (Fig. 1): (i) cell type-selective internalization of therapeutic enzymes, which includes delivery of toxic enzymes to cancer cells and cell type-selective ERT; (ii) localized prodrug activation, termed antibody-directed enzyme prodrug therapy (ADEPT) for cancer; (iii) direct modification of target cell surfaces or extracellular spaces, including degradation of cancer-driving epitopes and excess bone pyrophosphate; and (iv) delivery across biological barriers, such as the blood–brain barrier (BBB) for targeting of ERT to the central nervous system. Here we discuss the design principles and mechanisms of actions of each of these modalities and how they are used in different disease areas. Different approaches are employed to treat cancer *versus* other diseases, so these are discussed separately.

## 2. History and limitations of untargeted enzyme therapies

Enzymes are powerful therapeutics that can catalyze highly specific reactions within complex biological mixtures, avoiding molecules that are structurally and chemically similar to the desired reactant. This specificity is sometimes further tuned by cellular conditions, such as enzymes that are pH sensitive or rely on different cofactors for activity. Given their inherent catalytic nature, enzyme therapeutics can be effective when as little as a single copy is delivered to the cells of interest.

Enzymes are old therapeutic interventions, starting with digestive aids (such as amylase and pepsin) in the late 19th century^7^ and pancreatic enzymes (such as trypsin) for cancer therapies in the early 20th century.^8,9^ Enzymes from diverse biological sources continued to be used to treat a wide variety of disease until 1962,^8^ when the Kefauver–Harris Amendments to the Federal Food, Drug, and Cosmetic Act were passed. These amendments required therapeutics to be effective in addition to passing the prior safety requirements, establishing the groundwork for the modern United States Food and Drug Administration (FDA) approval system.^10^ This understandably and correctly limited the number of approved enzymes. The introduction of modern recombinant DNA technology and heterologous protein expression in 1977 further simplified production of therapeutic enzymes.^11^ This work came to fruition in 1987 with the first FDA approval of a recombinant enzyme drug, Alteplase (brand name Activase, Genetech).^12,13^ Now many therapeutic enzymes consist of recombinant human enzymes that are used to treat lysosomal storage disorders (LSDs).^8^

In 1966, the concept of ERT was first proposed, in which an exogenous enzyme is administered to replace a missing or mutated version.^13,14^ Since the Kafuever–Harris Amendment required expensive clinical trials, most pharmaceutical companies focused on large disease populations, and thus rare diseases that could be treated by ERT were often ignored. This changed with the Orphan Drug Act in 1983, which provided incentives – including market exclusively, tax credits and grants, and fast-tracking of development and approval – to companies to develop therapies for diseases affecting fewer than 200 000 people,^15^ thereby facilitating the development of enzyme therapies for rare diseases.^16^ In 1990, Pegademase (brand name Adagen, Leadiant Biosciences), became the first FDA approved ophan drug designated therapeutic enzyme. It is a modified form of bovine adenosine deaminase used to treat severe combined immunodeficiency caused by adenosine deaminase deficiency.^12,13^

Since the 1990s, there has been continual use and FDA approval of novel enzyme therapeutics, but they still only make up less than 15% of the currently licensed FDA-approved biologics (manually validated October 2022, per the FDA Purple Book).^17^ Some of the main factors that limit their expanded use are high levels of immunogenicity – discussed later – and inability to distinguish between substrates in diseased cells *versus* healthy tissue.^18,19^ The latter problem can be addressed with targeting.

The earliest and most used targeting modality is mannose-6-phosphate (M6P), which binds the cation-independent mannose-6-phosphate receptor (CI-M6PR) to traffic proteins to the lysosome. This modification is used in cellular homeostasis to retain lysosomal digestive enzymes but can be hijacked for lysosomal delivery of recombinantly expressed enzymes.^20^ Therefore, M6P is commonly used to target ERTs to the lysosome. To date, there are no known comparable methods to target enzymes specifically to other subcellular locations, such as the nucleus or cytosol. While not discussed more here, this is an unsolved problem limiting delivery of therapeutics more broadly, and there are ongoing efforts to address better delivery methods.^21^

There are currently four FDA approved enzymes that are targeted to specific organs and cells. These therapeutics use antibody and antibody fragments, other proteins (such as cytokines), and peptides (such as deca-aspartate) as targeting agents (Table 1). Preclinical studies and clinical trials have explored a wider range of targeting epitopes and encapsulation methods, including nanoparticles, erythrocytes, hydrogels, liposomes, and extracellular vesicles.^18,19^ As discussed in the sections below, different techniques are used against cancer, in which off-targeting is often toxic, as compared to targeted ERT, in which off-targeting is often non-toxic but limits efficacy to desired cells.

**Table 1:** FDA approved targeted enzyme therapies. All data from FDA Purple Book (downloaded October 2022),^17^ DrugBank,^12^ and company websites

Proprietary name	Ontak	Strensiq	Lumoxiti	Elzonris
Proper name	denileukin diftitox	asfotase alfa	moxetumomab pasudotox-tdfk	tagraxofusp-erzs
Company	Eisai Co.	Alexion Pharmaceuticals, Inc	AstraZeneca	Stemline Therapeutics, Inc
FDA approval year	1999	2015	2018	2018
Enzyme	Diphtheria toxin	Human tissue-nonspecific alkaline phosphatase	*Pseudomonas* exotoxin A	Diphtheria toxin
Targeting moiety	Interleukin-2	Deca-aspartate	Anti-CD22 disulfide stabilized variable fragments	Interleukin-3
Disease	Cutaneous T-cell lymphoma	Perinatal/infantile and juvenile onset hypophosphatasia	relapsed or refractory hairy cell leukemia	blastic plasmacytoid dendritic cell neoplasm
In use?	Discontinued due to production issues	Yes	Yes	Yes

## 3. Targeting enzymes to cancer cells and the tumor microenvironment

Cancer cell surfaces are often distinct from healthy cells. These changes can drive cancer progression through overexpression of immune inhibitory motifs (*e.g.* sialic acids and PD-L1),^22,23^ metastasis drivers (*e.g.* integrins),^24^ and receptors that initiate proliferation and anti-apoptosis signals (*e.g.* HER2).^25^ Therapeutically, these markers are used as targeting epitopes for antibody–drug conjugates (ADCs), checkpoint inhibitors, and targeted enzyme therapeutics.

There are three distinct methods to treat tumors with targeted enzymes: enzyme-based immunotoxins, ADEPT, and targeted delivery of cell surface-modulating enzymes. The first two methods have been extensively discussed elsewhere,^26,27^ so this review will focus primarily on their design principles and how they differ from the most similar non-enzymatic approach, ADCs. Immunotoxins and ADEPT bind to cancer cell-specific epitopes, but their mechanism of action is dependent on the eventual internalization of the construct or activated small molecule. The third class has not been discussed much elsewhere and offers a promising new alternative, in which changes on cancer cells serve as both the targeting moieties and the enzymatic targets.

### 3.1 Enzyme-based immunotoxins

**Fig. 2:**
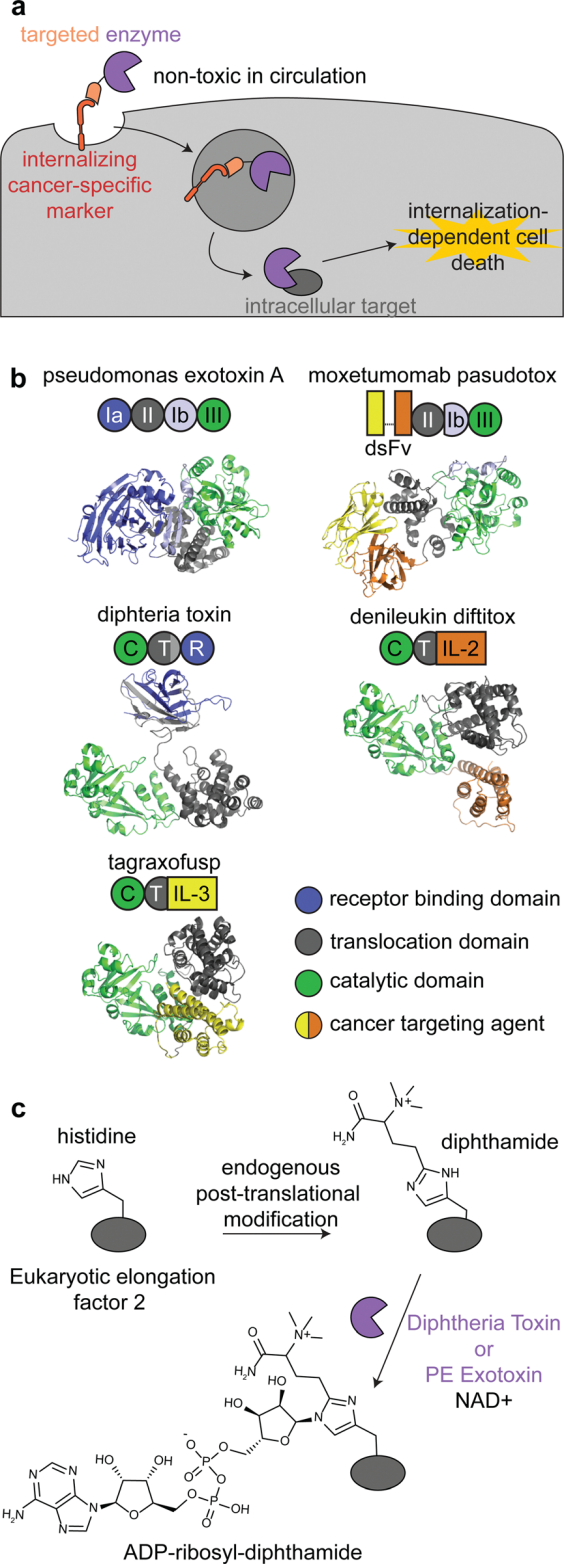
Mechanism, structures, and reactions of enzyme-based immunotoxins. (a) Overview of the mechanism of immunotoxin therapy, in which an enzyme with selectively for an intracellular target is internalized *via* a cancer cell-specific marker. (b) Schematics and structures of the three FDA approved immunotoxins which contain either *Pseudomonas* exotoxin A (PDB: 1IKQ) or diphtheria toxin (PDB ID: 1TOX). The structures of the immunotoxins were modeled by ColabFold^126^ using sequences obtained from DrugBank.^12^ Domains are labeled consistent with field standards and colored according to function. (c) ADP-ribosylation of a unique diphthamide posttranslational modification of eukaryotic elongation factor 2 by immunotoxins.

Immunotoxins are fusion proteins comprised of (i) an antibody or other protein that specifically binds to cancer cells, and (ii) a cytotoxic protein that kills the cells upon its internalization.^28^ This approach directly parallels ADCs, except an enzyme is delivered rather than a small molecule. Therefore, like with ADCs, immunotoxins are designed to bind to target receptors with increased expression on cancer cell surfaces *versus* healthy tissue, and binding to the receptor triggers internalization.^29^ To minimize on-target off-tumor toxicity, enzymatic activity must rely on internalization, and the enzyme should be non-toxic while circulating the body or adhering to off-target cell surfaces. This can be accomplished with an enzyme that is activated in the endosomal-lysosomal pathway and/or one that acts specifically on intracellular ligands (Fig. 2a).

There are currently three FDA approved enzyme-based immunotoxins: moxetumomab pasudotox (brand name LUMOXITI, AstraZeneca) for relapsed or refractory hairy cell leukemia, denileukin diftitox (brand name ONTAK, Eisai Co.) for cutaneous T-cell lymphoma, and tagraxofusp (brand name ELZONRIS, Stemline Therapeutics) for plasmacytoid dendritic cell neoplasm (Table 1).^12^

Moxetumomab pasudotox is comprised of *pseudomonas* exotoxin A (PE) fused to an anti-CD22 disulfide-stabilized Fv antibody fragment, while both denileukin diftitox and tagraxofusp consist of diphtheria toxin (DT) fused to interleukin-2 and interleukin-3, respectively (Fig. 2b).^12^ The vast majority of clinical trials with immunotoxins have used PE and DT, but other enzymes have also been used, including ricin A^30,31^ and shiga-like toxin A^32^ for cancer and graft *versus* host disease.^27,33,34^

PE and DT have the same mechanism of action: inhibiting protein synthesis through irreversible modification of eukaryotic elongation factor 2 *via* the NAD-dependent ADP-ribosylation of the diphthamide residue (Fig. 2c).^33,35,36^ Diphthamide is a unique posttranslational modification of histidine only known to be on cytosolic eukaryotic elongation factor 2, which is consistent with the intracellular selectivity requirements mentioned above.^36^

The use of PE and DT instead of classic cytotoxic small molecules has led to distinct therapeutic design principles, benefits, and shortcomings. As with many biologics, both ADCs and immunotoxins can elicit immune responses and antidrug antibodies. However, this risk is higher with immunotoxins, because of the addition of a bacterial enzyme.^28,33^ This has limited clinical trial progression and even use of the FDA approved immunotoxins due to poor tolerability.^26^

PE- and DT-based immunotoxins inhibit protein synthesis, so delivery of even a single molecule of the enzymes can effectively kill quiescent, nondividing cells.^33^ This mechanism of action does not overlap with many chemotherapeutics, allowing for synergism with other therapeutics and increased efficacy for some difficult to drug cancers.^33^ This toxicity profile also increases the stringency for target receptors, since unlike most ADCs, immunotoxins can kill healthy cells expressing even low levels of target receptors.^33,37^ To minimize off-tumor internalization, immunotoxins have been engineered to remove the enzymes’ native cell binding and internalization domains (Fig. 2b).^38^ All immunotoxins still have off-target toxicity, most notably vascular leak syndrome, indicating the need for further engineering.^34,39^

Furthermore, the use of an enzyme allows for directed evolution and other mutagenesis strategies to optimize the construct, contrasting the structure–activity studies required for small molecules. An example of this is the engineering of PE based immunotoxins to treat mesothelioma. Early clinical trials (start dates 2000–2011)^40–42^ used SS1P, which was comprised of an anti-mesothelin disulfide-stabilized Fv antibody fragment fused to the same PE fragment from moxetumomab pasudotox.^43^ Recent clinical trials (start dates 2016–2023)^44–47^ have used LMB-100, which is a de-immunized variant of the toxin, engineered from SS1P though T-cell and B-cell epitope mapping, point mutagenesis, and domain deletions.^43,48,49^ One final benefit of enzyme-based immunotoxins is that they can be expressed as single fusion proteins, simplifying production as compared to a biologic chemically conjugated to a synthesized small molecule.

### 3.2 ADEPT

**Fig. 3:**
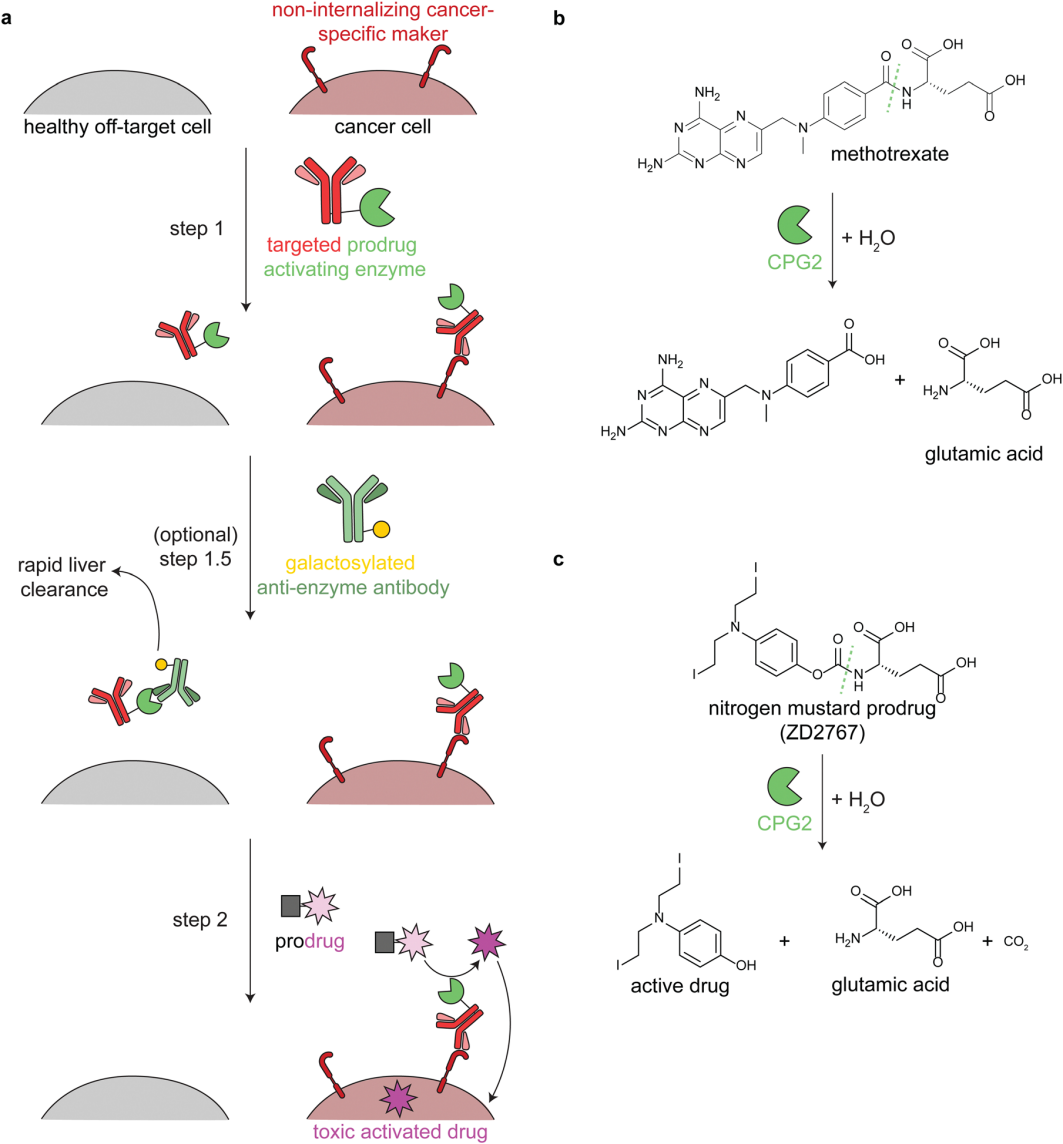
Mechanism and reactions of antibody-directed enzyme prodrug therapy. (a) Schematic showing the 2–3 steps in ADEPT therapy. (b) Reaction of untargeted FDA-approved carboxypeptidase G2 (CPG2) in cleaving excess circulating methotrexate in renal dysfunction. (c) Reaction of targeted CPG2 in activating a nitrogen mustard prodrug in cancer therapy. Prodrug shown (ZD2767) is one of the many similar prodrugs that have been used in clinical trials.^59,62^

Antibody-directed enzyme prodrug therapy uses an exogenous enzyme to activate a cytotoxic small molecule in the tumor microenvironment. This approach involves a minimum of two distinct steps. First, a prodrug-activating enzyme is targeted to cancer cells *via* an antibody against a cancer-specific non-internalizing receptor. The enzyme is selected or engineered to not catalyze extracellular reactions within a person other than prodrug activation. In the second step, a non-toxic prodrug is administered, where it is specifically activated in the tumor microenvironment by the antibody-enzyme conjugate. The activated drug is taken up into nearby cells, killing them (Fig. 3a).

Antibodies have long circulation times; for example the average half-life of trastuzumab (an anti-HER2 antibody) in clinical trials was 28.5 days.^50^ While generally a benefit therapeutically, excess circulating antibody-enzyme conjugates need to be cleared prior to prodrug administration. In clinical trials, this has been accomplished by specific glycosylation of the antibody-enzyme conjugate^51,52^ or even by adding a step prior to prodrug delivery, in which the patient is treated with a galactosylated anti-enzyme antibody for rapid liver clearance (Fig. 3a, step 1.5).^52–54^ For example, the same antibody-enzyme conjugate was tested in two different colorectal carcinoma phase I clinical trials, but only one of these trials included a clearance step. At the time of prodrug administration, the median tumor : blood ratio of the enzyme was 0.4 : 1 without the clearance step^55^ and >10 000 : 1 with the clearance step.^56^

ADEPT is proposed to solve delivery challenges of ADCs.^53,57,58^ In ADCs, the entire biomolecule is endocytosed, and a small amount of the activated drug escapes the lysosome. This is an inefficient process that is still being optimized. In ADEPT, only the activated drug needs to be internalized, a far easier cytosolic delivery challenge. However, extracellular activation of the prodrug increases the risk of off-tumor toxicity through toxic drug uptake into nearby non-cancer cells or even leakage of the toxic drug out of the tumor microenvironment. Therefore, prodrugs have been designed such that the corresponding activated drugs have short half-lives.^53,59^

Multiple enzymes from human and non-human sources have been used in ADEPT systems evaluated in preclinical studies, but only carboxypeptidase G2 (CPG2) from *Pseudomonas* sp. has been evaluated in clinical trials.^27,53,58,60^ CPG2 cleaves reduced and non-reduced folates, and a non-targeted version was approved in 2012 (brand name Voraxaze, BTG International Inc.) to cleave excess circulating methotrexate in renal dysfunction (Fig. 3b).^61^ This enzymatic activity has been repurposed in ADEPT systems to activate nitrogen mustard l-glutamate prodrugs (Fig. 3c).^27,56,57,59,62,63^

Like with immunotoxins, all ADEPT systems are immunogenic. Antidrug antibodies have been observed in all CPG2 ADEPT clinical trials, limiting the drug to one dose.^53,55,56^ However, repeated dosing of CPG2 ADEPT has been tolerated with co-administration of the immunosuppressive agent cyclosporine A.^64^ In an orthogonal approach, de-immunization through B-cell epitope mapping and mutagenesis of the antibody-enzyme conjugate yielded an ADEPT system that elicited antidrug antibodies in only 23% of tested patients,^65^ as compared to the 97% of patients observed with the non-engineered construct.^55,56^ Despite these limitations, ADEPT offers a unique approach to targeted delivery of cytotoxic small molecules.

### 3.3 Modulation of the cancer cell surface and tumor microenvironment

The third class of cancer-targeted enzyme therapies has distinct design principles and mechanisms of action as compared to the previous two. In this approach, a non-toxic enzyme is targeted to tumors, where it is not internalized but rather directly modifies the cell surface. This allows for altering of cancer surface markers that are difficult to drug with other approaches.

**Fig. 4:**
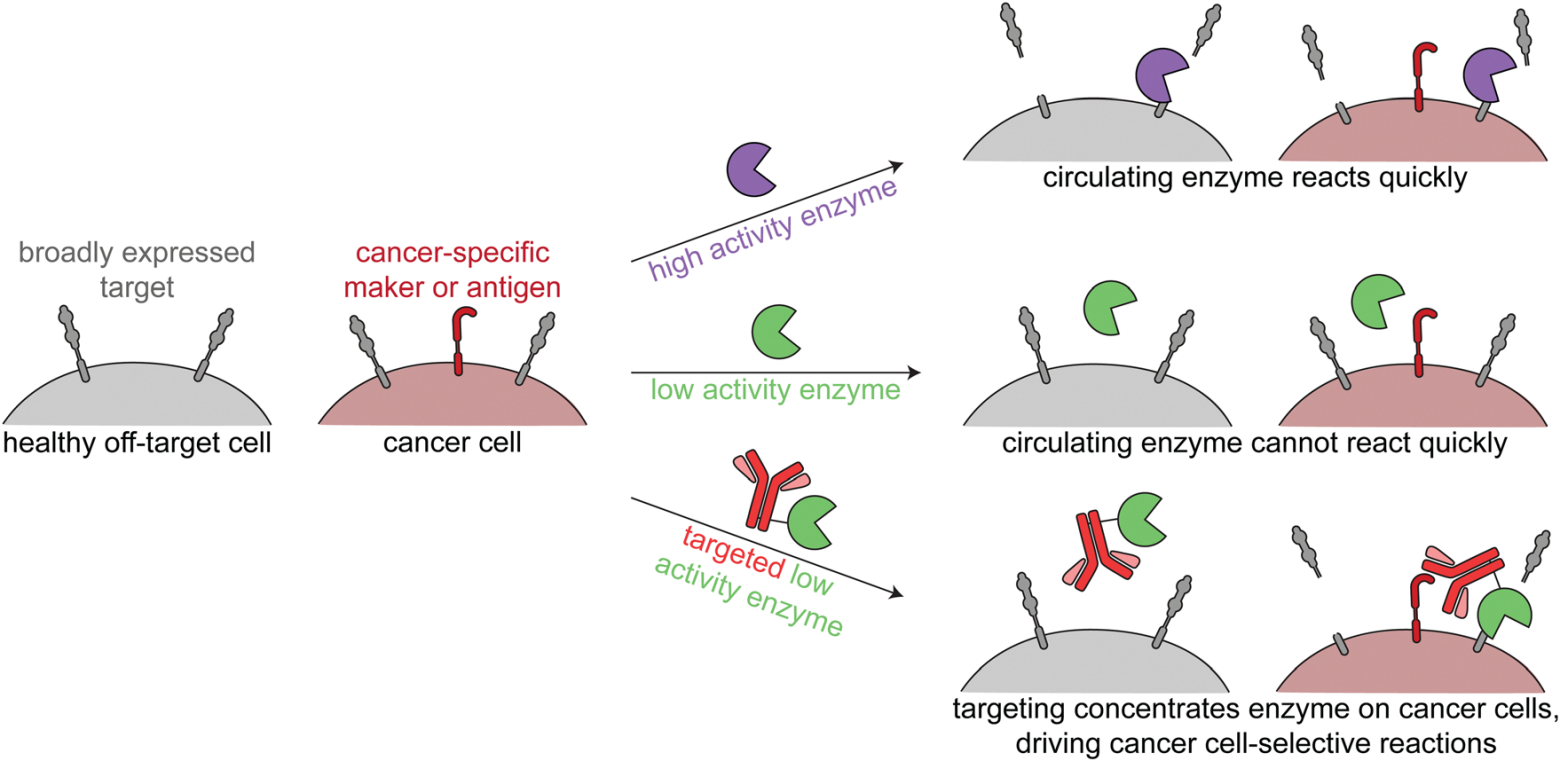
Overview of enzymatic modulation of cancer cell surfaces. Schematic demonstrating the need for a targeted low-activity enzyme to avoid on-target off-tumor reactions.

Since these enzymes act upon endogenous cell surface markers, there is a risk for deleterious on-target off-tumor activity. Relying on the principles of proximity induced reactions, low activity enzymes with weak substrate binding are optimal for this targeted approach. Such enzymes are minimally active when circulating the body, but when the enzymes are targeted to cancer cells, the high local molarity of substrates drive a pseudo-intramolecular reaction. Therefore, these enzymes need to be selected or engineered such that binding to cancer cells through the targeting agent drives the enzymatic reaction, rather than intrinsic binding of the enzyme to free substrate (Fig. 4).

Work from our group and others has focused on two main cancer targets, sialic acids and mucins, but these approaches are applicable to other substrates.

#### 3.3.1 Targeted sialidases

**Fig. 5:**
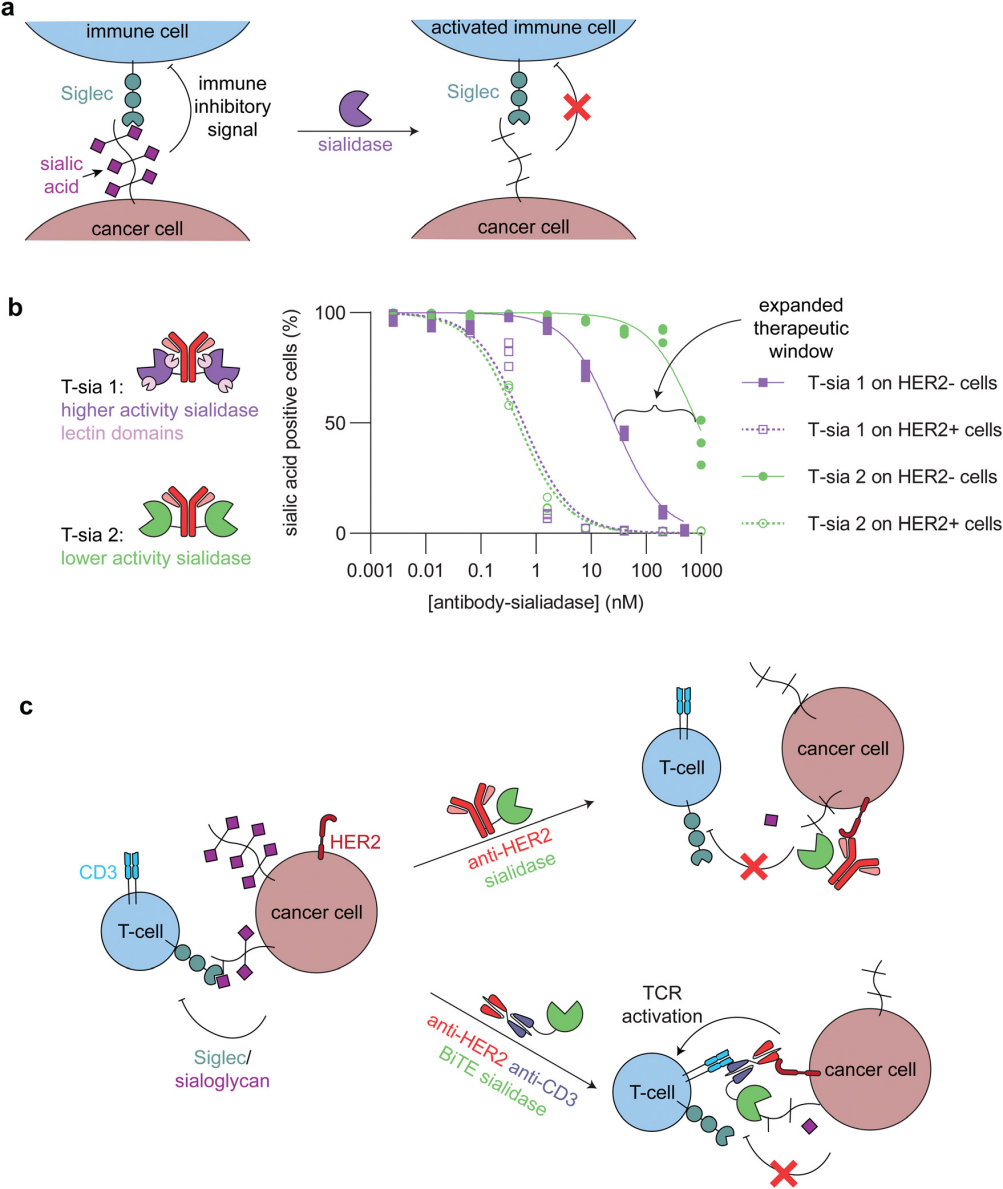
Targeted sialidases. (a) Schematic showing how sialidase treatment of cancer cells undermines the immune inhibitory signaling between sialic acids and Siglecs. (b) (left) Cartoons of the two different anti-HER2 antibody-sialidase conjugates. T-sia 1 used a higher activity sialidase with two lectin domains, which promoted non-antibody mediated binding to cell surfaces. T-sia 2 used a lower activity sialidase lacking lectin domains. (right) Dose-dependent sialic acid removal by T-sia 1 and T-sia 2 on HER2+ and HER2− cell surfaces as evaluated by flow cytometry (replotted using publicly available data from Gray *et al.*).^5^ The difference between off-target sialidase activity (removal of sialic acids on HER2− cells) with T-sia 1 and T-sia 2 represents an expanded therapeutic window for tumor-specific sialidase activity. (c) Schematic showing two of the different ways in which sialidases have been targeted to cancer cells. (*top*) An antibody-enzyme conjugate broadly desialylates cancer cells, which leads to increased immune cell killing through blocking of the immune-inhibitory Siglec/sialoglycan axis. (*bottom*) A bispecific T-cell engager (BiTE)-enzyme conjugate removes sialic acids at the immune synapse, promoting enhanced killing of the cancer cells through additional T-cell activation compared to the BiTE itself.

Sialidases (also known as neuraminidases) are enzymes that remove sialic acids, or negatively charged monosaccharides commonly appended to the end of glycan structures, exposing underlying sugars.^66^ These sialoglycans have distinct functions and signaling networks based on their structure and the linkage of the sialic acid to the rest of the glycan chain, but they generally act as immune inhibitory motifs through binding to sialic-acid-binding immunoglobulin-like lectins (Siglecs) on immune cells, paralleling classical immune checkpoints.^67^ A common phenotype of cancer is hypersialylation of different glycoconjugates, cloaking the tumor from immune cell killing (Fig. 5a).^23^ There have been multiple approaches to target sialidases to the tumor microenvironment, including antibody-enzyme conjugates,^5,68^ bispecific T-cell engagers (BiTEs),^69,70^ and chimeric antigen receptor (CAR) T-cells.^71^

Antibody-sialidase conjugates were designed to cleave the immune inhibitory sialic acids specifically from cancer cells, thus promoting immune cell killing of the desialylated cancer cells. In our first-generation antibody-sialidase conjugate (T-sia 1), *Vibrio cholerae* sialidase was conjugated to an anti-HER2 antibody (Fig. 5b, left).^68^ T-sia 1 removed sialic acids and Siglec-7 and Siglec-9 ligands from multiple cell lines and promoted natural killer cell-mediated antibody-dependent cellular cytotoxicity. However, T-sia 1 also desialylated non-target cells at moderate nanomolar concentrations, likely due to the relatively high activity of this sialidase on cell surfaces and the non-specific targeting of the enzyme to these surfaces through its lectin domains.^68,72^

We further optimized the antibody-sialidase conjugate with the generation of T-sia 2, which has lower off-target activity and higher chemical stability for *in vivo* use as compared to T-sia 1.^5^ This antibody-enzyme conjugate used a less active *Salmonella typhimurium* sialidase that lacks the previously mentioned lectin domains (Fig. 5b, left).^72^ The reduced activity and nonspecific binding expanded the therapeutic window of desialylation on only HER2+ cells and not HER2− cells from 60-fold (T-sia 1) to 2000-fold (T-sia 2) (Fig. 5b, right).^5^ This is consistent with the design principles mentioned above, indicating the importance of balancing activity with targeting.

Targeted sialidases can also reduce tumor burden in *in vivo* cancer models, as was observed with T-sia 2 delaying HER2+ tumor growth in a mouse breast cancer model.^5^ Follow-up *in vivo* work used a further optimized antibody-enzyme conjugate from Palleon Pharmaceuticals (E-301), comprised of two *Salmonella typhimurium* sialidase domains fused to the anti-HER2 antibody, trastuzumab. In multiple mouse tumor models, E-301 activated the adaptive immune system, repolarized tumor-associated macrophages, increased immune cell infiltration, and synergized with classical check-point blockade strategies.^73^ Further optimization of the antibody–enzyme conjugate by Palleon Pharmaceuticals led to E-602, which is currently being investigated in phase 1/2 clinical trials against a variety of different cancers.^74^

Sialidases have also been targeted specifically to the tumor-immune cell synapse through BiTEs, or bispecific antibodies that bridge a cancer cell and a T-cell and thus enhance T-cell killing of the target cancer cell, and CAR T-cells (Fig. 5c). Yang *et al.* and Szijj *et al.* both demonstrated that conjugation of sialidases to BiTEs enhanced T-cell killing of the target cancer cells as compared to unconjugated BiTEs.^69,70^ Yang *et al.* further evaluated the efficiency of BiTE-sialidases in controlling tumor burden in two cancer xenograft mouse models in immune deficient mice injected with human peripheral blood mononuclear cells and one syngeneic mouse model of melanoma. In all three models, the BiTE-sialidase reduced tumor burden as compared to vehicle control and the BiTE alone.^69^ In an orthogonal approach, Durgin *et al.* engineered CAR T-cells to secrete sialidase when activated by tumor cells. In multiple mouse tumor models, sialidase secreting CAR T-cells extended mouse survival and reduced tumor burden as compared to non-sialidase secreting CAR T-cells.^71^

#### 3.3.2 Targeted mucinases

We and others have applied the lessons learned from targeted sialidases to specifically degrade mucins on cancer cells using targeted mucinases. Mucins are a class of densely *O*-glycosylated glycoproteins that are commonly upregulated in cancers and have been shown to drive tumor progression through multiple immune-inhibitory and biophysical mechanisms.^75^ Mucins are difficult to drug with classical therapeutic and other bifunctional approaches, because they are a protein class comprised of repeated peptide domains highly modified with variable, branching, non-genetically encoded, and biosynthetically complex glycans, leading to an extended “bottle brush” conformation that can alter signaling and membrane biophysics.^76,77^

**Fig. 6:**
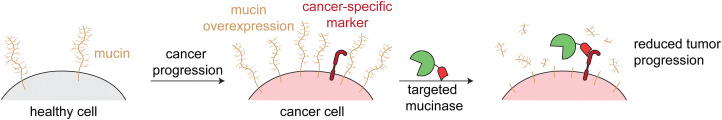
Targeted mucinases. Schematic showing the upregulation of mucins on cancer cell surfaces. Targeted cleavage of mucins *via* an engineered and targeted mucinase reverses mucin-driven tumor progressive pathways.

Mucinases, or bacterial mucin-selective proteases, offer a unique approach to specifically degrade mucins.^78,79^ Paralleling the antibody-sialidase work, we fused an engineered mucinase to an anti-HER2 nanobody to specifically degrade mucins on cancer cells. To avoid toxic on-target off-tumor mucin cleavage, the highly active enzyme was engineered to have reduced activity and binding to cell surfaces, consistent with the design principles demonstrated above for targeted sialidases. The resulting conjugate degraded mucins specifically on HER2+ cells, potentiated natural killer cell killing of only target cells, and reduced metastasis and primary tumor burden in multiple HER2+ mouse tumor models (Fig. 6).^6^

Similar to the CAR T sialidase work, Park *et al.* tethered a mucinase to a natural killer cell line and a chimeric antigen receptor expressing natural killer (CAR-NK) cell line. The tethered mucinase disrupted the mucin barrier at the interface between the immune and target cancer cells, which lead to increased target cell killing.^80^ Consistent with the effectiveness of the sialidase secreting CAR T-cells, this mucinase-tethered CAR-NK cell work demonstrates the potential of improving cellular therapies with targeted enzymes.

Sialic acids and mucins only represent a small fraction of targets that could be specifically altered by directing exogenous enzymes to cancer cell surfaces. These approaches therefore provide a blueprint for more broadly modifying the altered cell surface microenvironment in cancer.

## 4. Targeting to other organs/cell types

In contrast to many therapeutics that target cancer cells, targeted enzymes for other disorders are generally less toxic. The targeting is used to enhance delivery to organs that are either most affected by the disease or least treated with other therapeutics. Therefore, targeting can be less selective, and the enzyme is not required to be engineered to limit off-target activity. This approach is being used to transport enzymes across the BBB and to organs such as bones and muscles.

### 4.1 Delivery across the blood–brain barrier

**Fig. 7:**
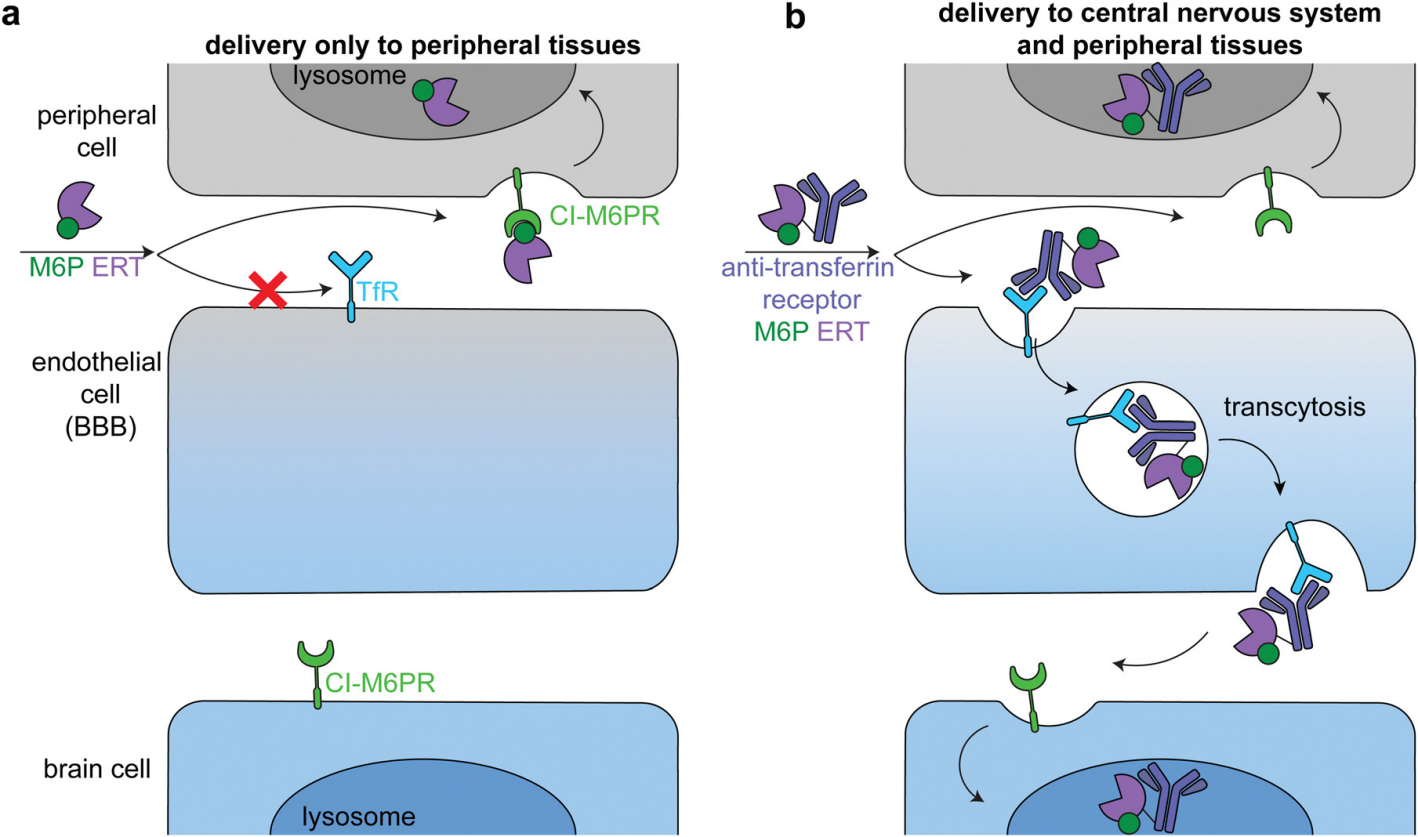
Delivery of therapeutic enzymes across the blood brain barrier. (a) An untargeted enzyme replacement therapy (ERT, purple) cannot cross the BBB, but is readily taken up into peripheral cells through binding of mannose-6-phosphate (M6P, *green*) on the enzyme by the broadly expressed lysosomal-targeting cation-independent mannose-6-phosphate receptor (CI-M6PR, green). (b) Endothelial cells express receptors, such as the transferrin receptor (TfR, dark blue), that can transcytosis cargo across the BBB. Fusion of an anti-TfR antibody (blue) to the M6P ERT delivers the enzyme to the central nervous system (CNS). The enzyme is endocytosed into both peripheral and brain cells through CI-M6PR trafficking of the M6P-modified enzyme to the lysosome.

Lysosomal storage disorders are characterized by enzyme deficiencies that lead to a toxic buildup of undegraded biomolecules in the lysosome. The frontline treatment for many LSDs is ERT, in which the missing enzyme is administered to restore wild type cellular function. However, many LSDs have pronounced neurological symptoms and pathophysiologies, and enzymes injected intravenously cannot readily cross the BBB to reach the central nervous system (Fig. 7a).^81^

The transportation of biologics across the BBB is one of the most difficult delivery challenges. A wide variety of approaches have been investigated to deliver enzymes to the central nervous system.^81^ One successful approach is hematopoietic stem cell therapy. When administered at a young age, hematopoietic stem cell transplantation is the gold standard for addressing the neurological components of some LSDs, including severe Hurler syndrome.^82^ Another successful approach has been to target ERTs to receptors on brain capillary endothelial cells, causing receptor-mediated endocytosis and transcytosis across the BBB (Fig. 7b).^83–85^ Two receptors that have been used in this way are insulin receptor and transferrin receptor.

Anti-transferrin receptor or anti-insulin antibodies have been used in preclinical studies and clinical trials to successfully deliver multiple enzymes to the central nervous system, most prominently in the treatment of Mucopolysaccharidosis II (Hunter syndrome).^86–88^ This disease is characterized by mutations in iduronate-2-sulfatase, leading to heparan sulfate and dermatan sulfate deposits across the body.^89^ In a phase 2/3 clinical trial, treatment with an anti-transferrin receptor iduronate-2-sulfatase fusion reduced heparan sulfate and dermatan sulfate concentrations in the cerebral spinal fluid and improved neurocognition in 21 of the 28 patients.^90^ The fusion protein also had a M6P modification that facilitated broad lysosomal targeting^86^ and thus had similar delivery profiles to peripheral tissues as the untargeted FDA-approved enzyme.^90^

Other approaches beyond antibody targeting have been used to hijack endogenous receptor-mediated transcytosis pathways. For example, Del Grosso *et al.* functionalized poly-(lactide-*co*-glycolide) nanoparticles with brain targeting peptides, including transferrin. Galactosylceramidase was loaded into these nanoparticles and successfully delivered to the central nervous system in a mouse model of Krabbe disease.^91^

Unlike in the cancer therapeutics space, off-target delivery is not a problem for ERT targeting the brain, because peripheral tissues are also enzyme deficient and there is a therapeutic benefit in restoring wild type cellular function in these cells as well (Fig. 7b, top). However, it is important to balance delivery across the body to maximize therapeutic effect. These approaches can be applied to the delivery of other enzymes to treat the many LSDs with neurological components.

### 4.2 Improved delivery to non-central nervous system organs

Enzymes used in ERT are often functionalized with M6P for their efficient delivery to lysosomes *via* engaging CI-M6PR. This strategy is not effective for delivery of non-lysosomal enzymes or if the target cells do not express CI-M6PR at high enough levels for sufficient uptake. Recent work on therapeutics for hypophosphatasia and Pompe disease demonstrate approaches to address both limitations.

Hypophosphatasia is caused by a deficiency of tissue non-specific alkaline phosphatase, a cell surface protease with enhanced expression in bones, liver, and kidney. This enzyme cleaves extracellular inorganic pyrophosphate to generate inorganic phosphate, which is required for hydroxyapatite crystallization, the mineral component of bone and teeth.^92^ Hypophosphatasia is primarily a disease of defective bone and tooth mineralization, which can manifest as fractures, tooth loss, skeletal pain, and rickets.^92,93^ Injection of the untargeted ERT was not effective, so the field turned to bone targeting approaches.^94^

In a proof of concept study, Kasugai *et al.* demonstrated that attaching six aspartic acids (Asp)_6_ to a fluorophore caused the fluorophore to bind to hydroxyapatite *in vitro* and specifically targeted it to bones and teeth in rats.^95^ A similar targeting motif, (Asp)_10_, was conjugated to tissue non-specific alkaline phosphatase to specifically target it to bones *in vivo.* The fusion construct (Asfotase Alfa, brand name Strensiq; Alexion) was further engineered through removing the enzyme's C-terminal glycosylphosphatidylinositol-anchor and attaching the human IgGγ1 Fc domain.^94^ In a phase 2 clinical trial for patients under 5 years old comparing 39 treated patients with 48 historical controls, Asfotase Alfa improved survival to age 1 from 42% to 95% and to age 5 from 27% to 84%.^96^ In older children (6–12 years old) treatment lead to substantial skeletal muscle healing.^93^ Asftoase Alfa was approved by the FDA for treatment of infantile and juvenile onset hypophosphatasia in 2015 (Table 1).^12^

Recent work addressing delivery of lysosomal acid alpha-glycoside for Pompe disease attempts to overcome a different issue: targeting of ERT to lysosomes when M6P is not sufficient. The skeletal muscle manifestations of Pompe disease (glycogen storage disease type II) are not successfully treated by the standard ERT (algucoside alfa, brand name Lumizyme; Sanofi).^12,97^ This is because both skeletal expression of CI-M6PR is low and the standard GAA ERT is not functionalized with much M6P.^98,99^ There have been a few different approaches to overcome this issue, including increasing the modification of the ERT with M6P, switching to other CI-M6PR targeting ligands, and targeting the enzyme through an orthogonal receptor.

A second-generation acid alpha-glycoside ERT (avalglucosidase alfa, brand name Nexviazyme; Sanofi) was designed for enhanced CI-M6PR targeting and uptake through modification with synthetic M6P-bearing glycans.^100^ In a randomized, double-blind phase 3 trial comparing Nexviazyme to the standard of care Lumizyme, Nexviazyme lead to greater improvements in respiratory and motor muscle function, indicating improved skeletal muscle delivery.^101^ Nexviazyme was approved by the FDA in 2021 for the treatment of late-onset Pompe disease.^12^ Similar approaches to increase consistent targeting of acid alpha-glycoside to CI-M6PR include fusion to insulin-like growth factor II, another CI-M6PR ligand,^102^ or expression of this enzyme in a proprietary cell line that leads to higher M6P content.^103^

An orthogonal approach is to bypass CI-M6PR altogether. Baik *et al.* fused acid alpha-glycoside to an antibody against CD63, an internalizing receptor enriched on skeletal muscles.^104^ The fusion protein trafficked to lysosomes *in cellulo* in a M6P-independent manner and removed more glycogen in muscle tissues than the standard ERT in a mouse model of Pompe disease. This approach of targeting an enzyme to a cell type-specific internalizing receptor is already being used in the cancer space (see immunotoxin section above). However, as nicely demonstrated by Biak *et al.*, this modality of targeted enzyme therapeutics has great value in other tissues and cells. CD63 was selected for skeletal muscle delivery, because it was known to be expressed highly on skeletal muscles (necessary for uptake), minimally expressed in the liver (necessary to avoid rapid liver clearance), and traffics between the cell surface and lysosome (necessary for internalization and delivery to the lysosome).^104^ For similar approaches to target enzymes specifically to other tissues, it is important that the targeting agent and receptor are carefully selected.

## 5. The potential of non-human enzymes and addressing immunogenicity

Most FDA approved enzyme-based therapeutics are recombinant human enzymes.^8^ While there can be great benefit in targeting these to specific cells and organs of interest, there is even greater therapeutic potential if we look beyond human enzymes to those from other animals, bacteria, viruses, plants, and fungi. Even considering only bacteria from human microbiotas, there are enzymes that perform therapeutically-relevant reactions with previously unseen specificity, such as selectively converting blood group antigens, or degrading only densely *O*-glycosylated mucins.^79,105^ Therefore, enzyme-based therapies could expand not only the list of druggable targets but also the ways in which these therapeutics modify human biology.

One example of this is the direct modulation of host immunity. Microorganisms have evolved effector molecules, including enzymes, to alter host immune responses through interactions with the innate immune system, autophagy, complement proteins, cytokines and chemokines, adaptive immune system, and cellular death pathways.^106^ Two of many such examples include production of inflammasome-inhibiting enzymes and secretion of highly selective IgA proteases.^107,108^ Novel therapeutics that alter immune responses could be beneficial in treating autoimmune disorders and cancer, improving efficacy of vaccinations, and reducing immunity to other therapies.

The expanded use of non-human enzymes would come at a cost: immunogenicity. Almost every biologic on the market comes with the risk of eliciting anti-drug antibodies and immune reactions. However this risk is greater with foreign enzymes, especially those that remain in circulation or on cell surfaces instead of being quickly internalized.^82,109^ Techniques to deimmunize enzyme therapies fall into two categories: (i) reducing the immunogenicity of the enzyme itself or (ii) modulating the immune system during dosing.

One way to reduce the inherent immunogenicity of an enzyme is to modify the most immunogenic structures and features, such as was done the immunotoxin LMB-100. These features are identified through multiple techniques, including phage display, incubation with patient derived T- and B-cells, and even computational prediction algorithms.^48,49,110^ Another popular option is to shield the entire enzyme surface with modifications such as polyethylene glycol (PEG), reductive methylation, or polysialylation.^110^ This is exemplified by the FDA approval of both unPEGylated and PEGylated asparaginase from both *Escherichia coli* and *Erwina chrystanthemi* for the treatment of acute lymphoblastic leukemia.^12^ However, some patients also develop anti-PEG antibodies, so care must be taken with repeated administration of PEGylated foreign biologics.^111,112^

The second option is to not alter the enzyme itself and instead modulate the host immune system during treatment. For example, ADEPT and ERT clinical trials have used cyclosporine to broadly suppress the immune system during dosing.^53,113,114^ Another approach is to tolerize the immune system against the single novel agent rather than broadly suppressing it. For example, Selecta Biosciences developed rapamycin-encapsulated nanoparticles (ImmTOR) that when co-administered with immunogenic biologic therapies, induce tolerogenic dendritic cells and antigen-specific regulatory T-cells.^115^ In animal models, ImmTOR effectively mitigated antidrug antibody formation against different therapeutic biologics without affecting immune responses to unrelated antigens.^115^ ImmTOR is currently being tested in a phase 3 clinical trial for treatment of refractory Gout with uricase.^116^

## 6. Conclusions and outlook

This review has discussed the therapeutic potential of targeting enzymes to specific cells and organ systems, but similarly to many other modalities, the future of proximity-induced enzymes is targeting a single biomolecule. The current approaches only target at the cellular level, and the molecular substrates are dictated by inherent enzymatic specificity. For example, DT and PE immunotoxins specifically modify eukaryotic elongation factor 2 because of their inherent specificity for the diphthamide post-translational modification and not because of targeting or engineering. With this system, we cannot easily redirect this activity to different post-translational modifications or protein targets, seriously limiting the modularity of targeted enzyme therapies.

To more closely mirror the single target specificity of PROTACs and molecular glues, we need to build modular targeted enzyme systems, in which the substrate is exclusively dictated by the targeting agent and the chemical reaction exclusively dictated by the enzyme. Nature has already evolved such systems, which we are beginning to understand and manipulate for therapeutic purposes. The most powerful example is CRISPR/Cas9, in which we can easily redirect the DNA cutting capabilities of Cas9 to any sequence in our genome based on the identity of the guide RNA.^117^ CRISPR/Cas9 is inherently a proximity-induced enzymatic system, but it is also being used to target other enzymes to specific DNA sequences, including cytidine deaminase for base editing,^118,119^ reverse transcriptase for gene alteration,^120^ and DNA polymerase for mutagenesis.^121^

While not yet translated to clinical use, several academic studies have used multiple strategies to engineer enzymes that target individual molecules rather than, for example, the cells that they occupy. Such approaches include splitting the enzyme into two non-functional units that only combine when bound to the target of interest,^122^ engineering enzymes to have enhanced specificity for an amino acid sequence found in only one target protein,^123,124^ or fusing broadly-active enzymes to highly-specific antibodies with fast off-rates, which have enhanced cleavage of the target substrate relative to the untargeted enzymes.^125^

Despite these limitations, proximity-induced enzymes combine the power of current enzyme therapies with the single-cell specificity of current bifunctional approaches. This has so far led to four FDA approvals, which provide novel mechanisms to drug cancers and treat previously untreatable skeletal manifestations of enzyme deficiencies. As discussed in this review, these design principles and approaches can be applied to other disease areas, and hopefully shepherd in a new era of precision biologics.

## Author contributions

G. S. T. performed the literature search and wrote the manuscript. C. R. B. provided conceptualization and wrote the manuscript.

## Conflicts of interest

C. R. B and G. S. T. are co-inventors on a patent related to the topic of this review, which has been filed by Stanford University (docket no. STAN-1929PRV). C. R. B. is a co-founder and scientific advisory board member of Lycia Therapeutics, Palleon Pharmaceuticals, Enable Biosciences, Redwood Biosciences (a subsidiary of Catalent), OliLux Bio, InterVenn Bio, Neuravid, Firefly Bio, Valora Therapeutics, and GanNA Bio.

## Supplementary Material
